# An immersive virtual reality system for ecological assessment of peripersonal and extrapersonal unilateral spatial neglect

**DOI:** 10.1186/s12984-023-01156-1

**Published:** 2023-03-18

**Authors:** Daniel Perez-Marcos, Roberta Ronchi, Arthur Giroux, Fanny Brenet, Andrea Serino, Tej Tadi, Olaf Blanke

**Affiliations:** 1 MindMaze SA, Lausanne, Switzerland; 2 Laboratory of Cognitive Neuroscience, Brain-Mind Institute, Ecole Polytechnique Fédérale de Lausanne, Lausanne, Switzerland; 3 Laboratory for Behavioral Neurology and Imaging of Cognition, University of Geneva, Geneva, Switzerland; 4 MySpace Lab, Department of Clinical Neurosciences, University Hospital of Lausanne, Lausanne, Switzerland; 5 Neuro-X Institute, Ecole Polytechnique Fédérale de Lausanne, Lausanne, Switzerland

**Keywords:** Virtual reality, Assessment, Unilateral spatial neglect, Stroke, Visuospatial attention, Peripersonal neglect, Extrapersonal neglect, Immersive, Brain injury

## Abstract

**Background:**

Unilateral spatial neglect (USN) is a debilitating neuropsychological syndrome that often follows brain injury, in particular a stroke affecting the right hemisphere. In current clinical practice, the assessment of neglect is based on old-fashioned paper-and-pencil and behavioral tasks, and sometimes relies on the examiner’s subjective judgment. Therefore, there is a need for more exhaustive, objective and ecological assessments of USN.

**Methods:**

In this paper, we present two tasks in immersive virtual reality to assess peripersonal and extrapersonal USN. The tasks are designed with several levels of difficulty to increase sensitivity of the assessment. We then validate the feasibility of both assessments in a group of healthy adult participants.

**Results:**

We report data from a study with a group of neurologically unimpaired participants (N = 39). The results yield positive feedback on comfort, usability and design of the tasks. We propose new objective scores based on participant’s performance captured by head gaze and hand position information, including, for instance, time of exploration, moving time towards left/right and time-to-reach, which could be used for the evaluation of the attentional spatial bias with neurological patients. Together with the number of omissions, the new proposed parameters can result in lateralized index ratios as a measure of asymmetry in space exploration.

**Conclusions:**

We presented two innovative assessments for USN based on immersive virtual reality, evaluating the far and the near space, using ecological tasks in multimodal, realistic environments. The proposed protocols and objective scores can help distinguish neurological patients with and without USN.

**Supplementary Information:**

The online version contains supplementary material available at 10.1186/s12984-023-01156-1.

## Introduction

Unilateral spatial neglect (USN) is a debilitating neuropsychological syndrome characterized by the failure to report, respond to or orient towards stimuli presented to the side of space opposite to a brain lesion, i.e. the contralesional side [1, 2]. USN is more frequently associated with a unilateral brain lesion affecting the right hemisphere [3, 4], therefore causing neglect for the left part of space, even if right USN following left brain damage can be also found [5]. USN can also negatively influence motor recovery [6], increasing the severity of an existing motor impairment and posing a serious problem for the rehabilitation process. Thus, the presence of USN is associated with a worse functional outcome and residual deficits in daily living activities after the hospital discharge [7–10]. Therefore, a correct identification of neglect symptoms is very important in the early stage after the brain lesion.

The reported rate of occurrence of USN in brain-damaged patients is highly variable, ranging from 10 to 80%, “likely [reflecting the] differences in the sensitivity of the tests and batteries used to detect the disorder” [11]. As USN is a multifarious syndrome, neglect symptoms can affect different sectors of the space [12, 13]: personal neglect, in which neglect patients do not pay attention and/or represent their hemi-body; peripersonal neglect, referred to the space that is within the arm-reach (near space); extrapersonal neglect, referred to the space that is out of the arm-reach (far space).

Current standard clinical assessments to evaluate USN are based on paper-and-pencil or simple behavioral tasks, which assess peripersonal neglect and, to some extent, personal neglect. In typical paper-and-pencil tests, patients are required to cross out targets, to read or to perceive stimuli usually presented on a A4 (or A3) paper sheet on a table in front of them. Paper-and-pencil assessment tools suffer from several limitations: (i) they could be not sensitive enough, with only a few parameters being recorded (e.g., the number of targets found by the patient); (ii) they are not ecological, as they do not reproduce complex daily life tasks (i.e., they ask to look for targets printed on a paper sheet); (iii) they are limited to a small portion of the peripersonal space (i.e., the size of the paper sheet); (iv) they require time-consuming examinations and manual analyses. Complementary behavioral assessments, also called ‘functional’ or ‘ecological’, can evaluate patients’ ability to perform activities of daily living (e.g., Baking Tray Task [14] or Catherine Bergego Scale [15, 16]). However, these behavioral assessments often heavily depend on the examiner’s subjective evaluation.

All these limitations contribute to the wide variability in the reported USN incidence rate among brain-damaged patients and make it difficult for the monitoring of patients’ evolution over time. Importantly, patients may exhibit normal scores on paper-and-pencil tests and yet show signs of neglect and substantial impairments in activities of daily living [17] or computerized tasks [18]. Therefore, more sensitive and objective tools for USN assessment are needed.

In recent years, increasing attention and research have been devoted to virtual reality (VR) as a promising tool for assessment and rehabilitation of USN [19, 20]. These attempts have consisted either in translating traditional paper-and-pencil tasks into VR, or in designing innovative tests in a virtual space. The main advantage of VR is to present the patient with an ecological situation, mimicking daily life. Moreover, in VR it is possible to fully control the environment and design different levels of difficulty, allowing more sensitive and fine-grained assessment. Finally, therapists can automatically compute additional performance parameters (e.g., pattern of stimuli exploration, velocity) that are not available in paper-and-pencil tasks, which are mainly limited to accuracy scores. Despite this important potential, most of the current experimental assessments of USN in VR are still limited to the peripersonal space, computing similar parameters as the classical tests (collected targets, total time employed, omitted elements) but translated into a virtual environment [21, 22]. A few attempts to evaluate the extrapersonal space showed a virtual environment on a 2D computer screen [23] or in 3D using a head-mounted display (HMD) without head tracker [24], both settings restricting the task to a very limited portion of the space too. A potential way to overcome this limitation consists in using immersive VR (iVR) approaches. Along this line, Kim et al. used an HMD to reproduce a realistic and safe street crossing environment to evaluate the ability to safely cross a road of USN patients [25]. Other studies using HMD have investigated only selective aspects of spatial exploration. For example, some research focused on the relationship between visuospatial neglect and navigation, sometimes with a moving virtual environment, sometimes tracking patients' real walking movements and asking them to avoid obstacles [26, 27]. Others proposed interesting and sensitive tasks, but the virtual environments or the stimuli were abstract and not ecological or realistic [28, 29]. To explore the environment and resolve the tasks, most of these paradigms used a joystick to perform the exploration and/or to select and validate the targets, therefore lacking naturalistic interaction [27, 30].

Within this regard, in a recent review Cavedoni et al. concluded that the usability of such solutions is not properly evaluated and recommends careful evaluation when validating new solutions [31]. Therefore, despite recent progress in developing complex VR tools, some limitations persist in such tools, including lack of complete evaluations using naturalistic tasks reflecting daily life activities, task difficulty tailored to clinical contexts and extensive feasibility tests.

Here we present an innovative iVR-based battery for the assessment of extrapersonal and peripersonal USN, which allows 3D exploration and could potentially facilitate the transfer of recovery and rehabilitation effects to daily life activities. The conceptual innovation of our tasks lies in the inclusion of both near and far space realistic activities, including static and dynamic targets, in which the participant can freely explore the environment with the movement of the head as in real life and directly reach the targets with the arm effector in the near space task. In addition, both the scene and the targets reproduce real-life scenarios, and are not abstract. The aim of the study is therefore to describe the technical implementation of the proposed battery and assess its usability and feasibility testing a middle-age and elderly population of neurologically unimpaired participants. These scores can be used to define cut-off scores of the pathology for brain-damaged patients. The clinical validation of these tasks is beyond the scope of this paper.

## Materials and methods

### Participants

39 healthy adult participants with no history of neurological and/or psychiatric diseases (16 female; 49.44 ± 7.94 years, range of age: 39–70 years) were recruited by e-mail advertising. They all had normal or corrected-to-normal vision. All participants were right-handed (laterality index > 80%) according to the Edinburgh Handedness Inventory [32]. The study was carried out in accordance with the regulations of the Ethics Committee of the Ecole Polytechnique Fédérale de Lausanne, and all the participants gave a written informed consent. A compensation of 20 CHF/hour for their participation was attributed. Two participants could not perform the near space task due to technical problems (N = 37 for this task).

### Equipment

For both tasks we used an Oculus Rift DK2 (Oculus, Menlo Park, CA, USA) head-mounted display (HMD), with a nominal static field of view of up to 100°. We extracted head gaze information from the head tracking sensor from the HMD to update the environment (allowing the exploration of the virtual space) and to compute parameters related to space exploration. We used headphones for higher immersion into the virtual environments, and to deliver the sounds in the dual task. The 3D environments were generated using the game engine Unity (Unity Technologies, San Francisco, USA). Raw data were stored in CSV files. We generated statistics from the CSV files to spreadsheets using Python 2.7 scripts. The tasks ran on a PC with Windows 8.1, Intel i-7 core, 8 GB of RAM and a Nvidia Geforce GTX 980. In addition, for the near space task we used MindMaze’s proprietary motion tracking system, based on a stereo camera and markers for detection of upper body movements in the 3D space. The application included a simple graphical user interface that allowed the experimenter to enter subject data (ID number, gender, age, handedness), and to select and launch the level to be performed.

### iVR-based far space task for USN assessment

**Fig. 1:**
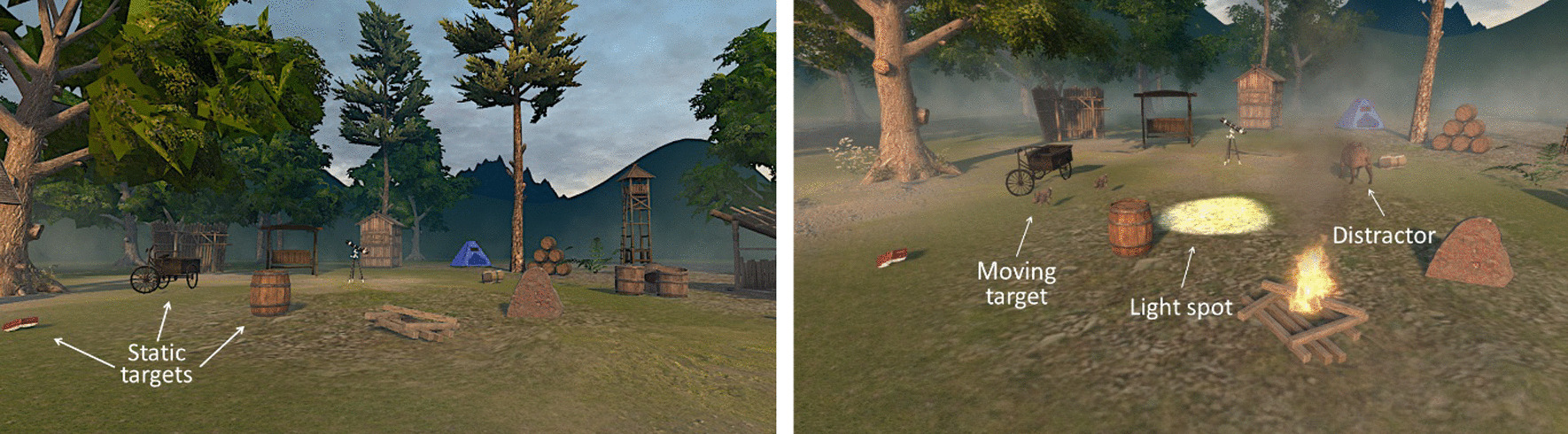
Virtual environment displayed to the participants via the HMD for the far space task (Level 1). Screenshot of the Level 3, with targets, distractor and light spot visible

Once participants wore the HMD, they were immersed in a 360° virtual forest composed of trees, plants and a set of objects (Fig. 1). The environment included ecological sounds from wind moving the leaves and a central fire for higher immersion. We divided the virtual space in six columns (L3-L2-L1-R1-R2-R3), covering the field of view from the extreme left (L3) to the extreme right (R3). All targets appeared from the participant’s position in the virtual environment onwards, i.e., in front of the participant (no target appeared behind the participant). To explore the extreme left and right spaces, the participant had to turn the head 90° towards the left or the right side, with respect to the alignment of the head to the mid-sagittal plan of the body.

We developed four sub-tasks with different levels of difficulty defined by the type of target (static versus moving), the absence or presence of distractors, and a single or dual task to be performed. Level 1 consisted in finding 18 static objects (horse, book, wooden house, bike, etc.) in the virtual forest. The environment also contained trees, plants and a fire. We instructed participants to consider them as part of the background (i.e., not a target). Targets were distributed at three different distance ranges from the participant’s viewpoint (computed at around 8–10, 14–18 and 25–36 m), i.e., all in the extrapersonal space, for each of the six spatial columns defined above. Participants were asked to tell the experimenter when they thought to be finished (there was no time limitation). Levels 2–4 consisted of finding in the scene 36 moving targets (rabbits), appearing in subsequent trials. In Level 2, there were only static distractors (the targets from Level 1, which were always displayed). In Level 3, a moving distractor was displayed in each trial and in addition to the target (the rabbit): small (chicken) or big (boar) distractors appeared in a different column, simultaneously to the targets (Fig. 1). Targets and distractors could appear either alone or in a group of two (e.g., one or two rabbits). In Level 4, we included an additional auditory dual (oddball) task to the Level 3, where participants had to report verbally when they heard a dog barking (versus a cricket, which sounded at 90% of times). A sound was played every second with a random interstimulus interval between 0.5 s and 1.5 s.

In all levels, targets were equally distributed along the six columns covering the whole space. Overall, 18 targets appeared in each of the hemispaces, half coming from the top, the other half from the bottom (i.e., from the front horizon or from the back side with respect to the participant viewpoint). In order to maximize the exploration of the space, we avoided displaying consecutive targets in the same column and coming from the same direction, and continuously varied the hemispace placement of targets, so that participants had to cross the midline in more than 60% of the trials. For example, if a rabbit came from the top of a column (and finishing on the bottom), the rabbit(s) in the next trial did not appear on the bottom of the neighboring column to avoid appearing just next to it, facilitating the exploration. Trajectories (column and direction) from both targets and distractors animals were equally distributed when jointly appearing. All these conditions translated into 18 left and 18 right distractors. Their apparition locations were as follows: if the rabbits appear on the bottom left, distractors appeared 6 times from the top left and 12 times from the right (6 from bottom and 6 from top); a swapped distribution occurred for top left rabbits. For top and bottom right rabbits, a mirrored distribution occurred. This distribution aimed at maximizing the number of required midline crossings in order to succeed in the task, while preventing participants to predict the appearance location of the next target. Regarding the type of distractors, boars (big distractor) appeared 10 and 9 times on the left and right sides, respectively; chickens’ (little distractor) distribution was 8 and 9, respectively).

Participants used the embedded head tracker in the HMD to control a pointer (Level 1) or a light spot (Levels 2–4) to select the targets in the scene, and the space bar of the computer keyboard to validate the selected item. Once the target selected, a green contour was displayed around the item to indicate that it had already been selected. In Levels 2–4, moving targets appeared trotting into the scene from the top (horizon) or the bottom (back side of participant’s viewpoint) of the image, and they stopped for 5 s in the middle of the scene at one of the three predefined positions (all in the field of view of the participant) before continuing their movement and disappearing from the scene: therefore, each target is displayed for a maximum time of 20 s. However, as soon as the participant validated a target, the targeted animal ran out of the scene quickly as the trial has been successfully terminated. Then the next trial started. Participants were not instructed about the number of targets in each level.

### iVR-based near space task for USN assessment

**Fig. 2:**
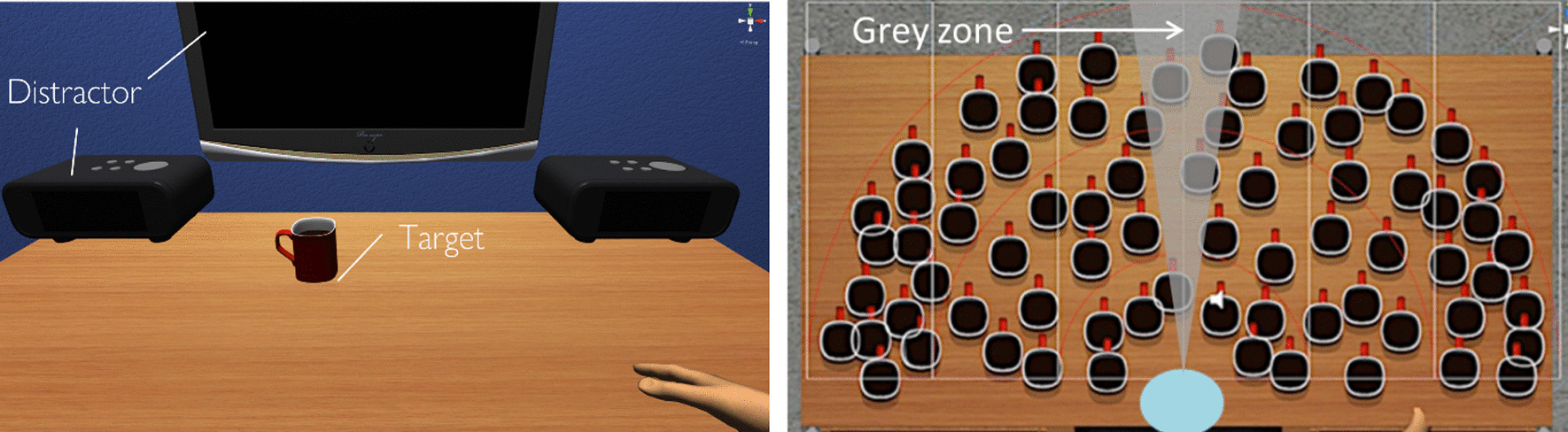
Virtual environment of the near space task, where participants see an avatar from a first-person perspective; mug distribution and start pad position (in blue). The grey area represents the neutral zone that it is considered as central, i.e., neither left nor right (see Results section for more details)

Once participants wore the HMD, they were immersed in a 360° virtual room composed of virtual lamps and a TV on the background, and a virtual table on which two clocks were placed on the top right and left corners (Fig. 2). Participants were embodied in a virtual avatar, seen from a first-person perspective, seated in a chair and in front of a virtual table. The avatar reproduced participants’ upper body movements in real time as captured by the motion tracking system.

We divided the space in six columns (L3-L2-L1-R1-R2-R3), covering from far left (L3) to far right (R3), and three egocentric semi-circles, all in the reaching workspace (Fig. 2). We developed three sub-tasks with different levels of difficulty defined by the absence or presence of distractors, and a concomitant auditory dual task.

In each trial, participants were required to find and reach a mug, that appeared at different locations on the table. Each level included 72 trials. Targets were equally distributed by column and semi-circles (4 for central ones (L1-R1), 6 for L2-R2, and 12 for L3-R3). The starting point of participants’ hand was in front of the mid-sagittal plan of their body, indicated with a virtual blue pad. As soon as a target appeared, they had to move towards and reach the mug. A five-trial testing phase was introduced before the assessment for participants to familiarize with the task. During the task, the target disappeared after the virtual hand had reached it (trial was considered as successful if the hand continuously stayed on the target for 0.5 s to encourage purposeful movements versus arbitrary exploration), or after twice the average time-to-reach computed during the testing phase (failed trial). In order to start the next trial, participants had to bring their virtual right hand to the central blue start pad, intended to reduce the bias in reaching times as participants used their right hand for the task.

Level 1 included no active distractor. Level 2 consisted in finding the mug in the presence of different lateralized (clock, lamps) or central (TV) distractors that appeared individually or by couples every 30 s in a pseudo-random order, equally distributed in the left and right hemispaces.

For the Level 3, we included an additional auditory dual (oddball) task to the Level 2, where participants had to report verbally when they heard a bell ringing (occurring 10% of times) versus a hammer hitting a table lightly (90% of times). An auditory stimulus was presented on average every second with a random interstimulus interval between 0.5 s and 1.5 s.

### Paper-and-pencil tasks

In order to compare the performance of participants in VR and in standard tests, all participants underwent to these standard paper-and-pencil tests:

- *Star cancellation* [33]: The patients’ task was to cross out all of the 56 black small stars (30 in the left-hand-side, 26 in the right-hand-side) printed on an A4 sheet, together with distracters (larger stars, letters, and words). The total number of targets’ omissions, and the difference Left vs. Right omissions were computed.
- *Line bisection* [34]: The patients’ task was to mark with a pencil the mid-point of six horizontal black lines (two 10 cm, two 15 cm, and two 25 cm in length, all 2 mm in width), presented in a random fixed order. Each line was printed on an A4 sheet, with the center of each line being aligned with the mid-sagittal plane of the subject’s body. The score was the deviation of the subjects’ mark from the objective midpoint, measured to the nearest mm; a positive score denoted a rightward displacement, a negative score a leftward displacement.
- *Copy drawing* [35]: Patients were required to copy a daisy printed in the center of an A4 sheet. The total omission of elements, and the Left–Right difference in the omission, was computed.

### Experimental procedure

Participants sat comfortably in front of a desk. For the far space task, a keyboard was placed on the desk for the participant to press the space bar with the right hand. For the near space task, we placed 6 colored passive markers on both shoulders, elbows and wrists of the participant in order to track the upper limb movements. Then, a two-step calibration procedure was made (i) to match participant’s arm with the virtual one, and (ii) to calculate the extension of the workspace, i.e., the maximum reaching distance of each participant, in order to place the virtual targets within each participant’s reachable space. For both iVR-based tasks, the virtual environments were re-centered at the beginning of each level, so that the center of the virtual environment was aligned to the body midline of the participants.

The study consisted of three blocks, with the participants performing the iVR-based far space, iVR-based near space, and paper-and pencil standard assessments in a counter-balanced order. For the iVR-based far and near space tasks, participants completed all levels in the order of increasing difficulty. Before starting each level, a short training was performed to familiarize participants with the corresponding task. For the standard assessments, participants completed the three paper-and-pencil tasks in a counter-balanced order. The sheets were placed centered with respect to the body midline. After each block of tasks, participants were debriefed and filled in a questionnaire about their experience. Participants completed each series of tests once.

### Performance variables

For the iVR-based far and near space USN assessments, a comprehensive set of parameters was extracted from participants’ responses, as well as the head and upper body movements. For instance, the time spent by the participant exploring the left and the right sides with respect to the body midline is automatically recorded, offering an objective measure to detect eventual asymmetries in participants’ spatial orientation. Table 1 lists the parameters obtained for each task. After each level of each task, we automatically generated spreadsheet files containing the computed variables as well as plots for immediate and intuitive overview by experimenters and therapists of participants’ performance.

**Table 1:** Main parameters of interest for each of the iVR-based tasks

	Far space	Near space
Number, position, and detection time of detected/omitted targets; L-R difference of omitted targets	X	X
Total and lateralized time of exploration, and L-R difference	X	X
Ratio of exploration time (time spent on the right (left)/number of detected targets on same side)	X	X
Time moving toward left and right direction (head gaze)	X	X
Lateralized average and standard deviation of marking distance and time (i.e., the distance or time between 2 successively detected targets, correspondingly; Level 1 only)	X	
Average and standard deviation of reaching time by column and side		X
Time required to reach a target (time-to-reach): time taken from target appearance to the moment the target disappears after being touched		X
Reaction time: time taken to move the hand ~ 2 cm in the direction of the target after target appearance		X

### Questionnaire

Before and after each of the three blocks of tasks (paper-and-pencil, and iVR-based far and near space), participants were asked to answer a set of questions regarding their experience (Table 2). The experimenter read the questions aloud to the participants, who were instructed to answer by pointing with their right hand on a seven-point vertical colored Likert scale (gradually changing from red to green; without numbers or text labels). The answers were scored linearly from 1 (‘completely disagree’) to 7 (‘completely agree’). Questions Q1–Q2 reported the levels of relaxation and fatigue, and were asked both before and after the assessments. The rest of the questions were asked after the assessments only. Q3 reported the perceived concentration level. Q4–Q6 gave information about the accuracy of the motion tracking system. Q7–Q9 measured the level of presence, body ownership and sense of agency in the virtual environments. Q10–Q11 referred to comfort issues known from iVR. Q12–Q16 provided insights about the appropriateness and parametrization of the tasks. Q17–Q19 evaluated levels of enjoyment and task preference.

**Table 2:** Scores (mean ± std) for the questionnaire items regarding paper-and-pencil and both iVR-based far and near space tasks. Scores range from 1 (‘completely disagree’) to 7 (‘completely agree’)

Question	Paper	Far	Near
**Fatigue, relaxation and focus**			
1. [Before the task:] Do you feel tired?	3.1 ± 1.9	2.7 ± 1.8	2.5 ± 1.7
2. [Before the task:] Do you feel relaxed?	5.7 ± 1.5	6.1 ± 1.2	5.8 ± 1.5
1b. [After the task:] Do you feel tired?	2.7 ± 1.9	3.7 ± 2.0	2.8 ± 1.7
2b. [After the task:] Do you feel relaxed?	6.4 ± 0.9	5.5 ± 1.8	5.4 ± 1.4
3. Were you focused on the task?	6.7 ± 0.6	6.8 ± 0.5	6.8 ± 0.4
**Accuracy of motion tracking system**			
4. When you were in the starting position on the blue circle, did the position of the virtual hand correspond with that of your own hand?			6.0 ± 1.0
5. When you attained the target, did the position of the virtual hand correspond with that of your own hand?			6.1 ± 1.0
6. Were the movements of the virtual arm synchronized with the movements of your own arm?			5.4 ± 1.6
**Presence, body ownership and sense of agency**			
7. During the task, did you have the feeling to be in the virtual environment?		6.4 ± 1.3	6.1 ± 1.2
8. Did you have the feeling that the virtual arm was your own arm?			4.5 ± 2.0
9. Did you have the feeling of controlling the movements of the virtual arm?			6.3 ± 0.8
**Comfort**			
10. Did the task make you feel physical discomfort?	1.0 ± 0.2	2.2 ± 1.7	1.6 ± 1.2
11. Did the task make you dizzy?		1.8 ± 1.5	2.5 ± 2.0
**Task design**			
12. Was it difficult to perform the task?	1.6 ± 1.1	2.8 ± 1.8	2.3 ± 2.1
13. Did you have the time to attain the targets?		6.6 ± 0.8	6.9 ± 0.2
14. Did you feel like you could attain the targets in the space?		6.4 ± 0.6	6.9 ± 0.3
15. [If dual task] Was the secondary task (the sound test) distracting?		3.2 ± 2.0	1.9 ± 1.4
16. [If dual task] Was it difficult to achieve both tasks?		3.4 ± 1.7	2.2 ± 1.4
**Enjoyment & preference**			
17. Was the task enjoyable?	6.1 ± 1.2	5.7 ± 1.3	4.6 ± 1.6
18. Was the task boring?	1.4 ± 1.1	2.7 ± 1.7	3.3 ± 2.0
19. Which task did you prefer?	7	29	3

### Statistics

Statistical parametric analyses were run on the data collected on the iVR tasks and the questionnaire. The analyses focused on finding differences in the reported scores (questionnaires, number of omissions, exploration times, etc.). Additionally, we also analyzed L-R differences for specific parameters, including exploration times and number of omissions. When data distribution did not fit the assumption for parametric analyses (Lilliefors test used), non-parametric analyses were run. The software used for the statistical analyses was MATLAB 18b (MathWorks Inc.). The significance level was set at p < 0.05.

## Results

We report in next subsections the results obtained for each task. No adverse event was reported.

### iVR-based far space task for USN assessment

The results of the far space task showed that in Level 1 (static targets), 26 out of 39 participants made their first selection on the left hemispace, with 19 of them selecting the nearest left column to the midline (L1). Participants spent 1 min on average to complete Level 1 of the far space task, between 8 and 10 min for each of the other levels (Additional file 1: Table S1).

The number of omitted targets was very low in each level (< 1 target omitted on average; maximum number of 4 omissions in a single level; see Additional file 1: Table S1), with no differences between the left and right omissions for Levels 1, 2 and 4 (Wilcoxon sign rank test; Level 1: Z = 0, p = 1; Level 2: Z = − 1.13, p = 0.257; Level 4: Z = − 1.43, p = 0.153). In Level 3, and despite the global number of omissions was very low, more omissions on the right than on the left hemispace were observed (Z = − 3.54, p < 0.001). When the dual task was included (Level 4), participants detected on average 98.1% of the targeted sounds, without the omission scores being affected.

Regarding the exploration time (i.e., the actual time spent on the left or right hemispaces), participants equally distributed their head gaze between the two hemispaces in Level 1 (Wilcoxon sign rank test: Z = 0.29, p = 0.769), while in Levels 2–4 (dynamic targets) we observed a statistically significant bias of about 55% (L) vs. 45% (R) exploration time distribution in all three levels (paired t-test; Level 2: t = 6.59, p < 0.001; Level 3: t = 6.97, p < 0.001; Level 4: t = 5.89, p < 0.001). To obtain an index of spatial processing assessing both exploration time and accuracy, we computed the ratio of exploration time (i.e., time spent on the right (left) divided by the number of cancelled targets on the same side), which represents a measure of task efficiency for each hemispace. Statistical analysis reported similar L-R differences to those of the exploration times (Wilcoxon sign rank test: Level 1: Z = 0.28, p = 0.780; Level 2: Z = 4.14, p < 0.001; Level 3: t = 5.49, p < 0.001; Level 4: Z = 3.44, p < 0.001). Additional file 1: Table S1 shows the detailed scores for each variable of interest.

The head tracker embedded in the HMD provides information about head gaze. Therefore, we investigated to which extent the head gaze reflects the perceptual exploration of the space. In order to estimate its value, we defined a neutral zone, i.e., a central space extending 10° (5° for each side) radially from the observer point of view, which was not considered neither left nor right hemispace.

We observed that participants spent 10% of the time within this zone in Level 1, and 18% in Levels 2–4. The distribution of the exploration times changed to (L)44% vs. (R)45% for Level 1 (Z = − 0.47, p = 0.635), (L)46% vs. (R)35% for Level 2 (t = 5.41, p < 0.001), (L)45% vs. (R)37% for Level 3 (t = 5.76, p < 0.001), and (L)44% vs. (R)38% for Level 4 (Z = 3.39, p < 0.001). The neutral zone did not alter the spatial time distribution significantly.

When considering the time spent moving towards a position on the right or the left spaces, we observed that participants spent the same amount of time moving their head towards both sides in Level 1 (Wilcoxon sign rank test: Z = 0.77, p = 0.443). However, a slight, but statistically significant, unbalance was observed in the other levels (Level 2: Z = − 2.71, p = 0.007, longer time to move towards the right side; Level 3: Z = 2.34, p = 0.019, longer time to move towards the left side; Level 4: Z = 2.29, p = 0.022, longer time to move towards the left side).

An additional parameter of global visual search efficiency was measured for Level 1. The average marking time (and average marking distance) reflects the average time taken to cancel next target (or the average distance between the two consecutive targets), which can be placed on the left or right part of the space with respect to the previous one (Additional file 1: Table S1). No difference was observed neither for the marking distance (paired t-test: t = 0.584, p = 0.561) or the marking time (Wilcoxon sign rank test: Z = 0.52, p = 0.606) towards the left and the right. We also assessed the variability of these parameters computed as the standard deviation. No difference was observed neither for the standard deviation of the marking distance (paired t-test: t = 0.527, p = 0.599) or of the marking time (Wilcoxon sign rank test: Z = 1.05, p = 0.295).

### iVR-based near space task for USN assessment

With respect to the near space task, the number of omitted targets was also very low (< 0.5 target omitted on average; maximum number of 3 omissions in a single level; see Additional file 1: Table S1), with no differences between the left and right omissions in Levels 1 and 3 (Wilcoxon sign rank test; Level 1: Z = 0.45, p = 0.655; Level 3: Z = − 1.89, p = 0.059) and a bigger number of omissions in the right hemispace in Level 2 (Z = − 2.31, p = 0.021). When the dual task was included (Level 3), participants detected on average the 99.2% of the targeted sounds, without the omission scores being affected. The duration of the task for each of the Levels 1–3 was between 4 and 5 min, depending on participant’s reaching time (Additional file 1: Table S1).

The analysis of the exploration time yielded higher exploration times for the left hemispace, with more heterogeneous values across levels (paired t-test; Level 1: t = 7.10, p < 0.001; Level 2: t = 7.61, p < 0.001; Level 3: t = 2.87, p = 0.005) (Additional file 1: Table S1). Given the low number of omissions, the ratio of exploration time (i.e., time spent on the right (left) divided by the number of cancelled targets on the same side) followed similar pattern as the exploration time (paired t-test; Level 1: t = 7.07, p < 0.001; Level 2: t = 7.58, p < 0.001; Level 3: t = 2.83, p = 0.006). The distribution of the exploration time was asymmetric, with left/right rates of 64%/36% (paired t-test: t = 7.10, p < 0.001) for Level 1, 67%/33% for Level 2 (t = 7.61, p < 0.001), and 57%/43% for Level 3 (t = 2.87, p = 0.005) (Additional file 1: Table S1).

In this task, we observed that participants preferably moved their eyes, and not their heads, since most objects appeared in their central, near- or mid-peripheral view. When including the analysis of the neutral zone at the center of the visual field (Fig. 2, right), we observed that participants spent 50.8% of the time within this zone, therefore affecting the distribution of the exploration times, which changed to (L)33% vs. (R)14% for Level 1 (t = 5.92, p < 0.001), (L)31% vs. (R)12% for Level 2 (t = 6.02, p < 0.001), and (L)24% vs. (R)17% for Level 3 (Wilcoxon sign rank test: Z = 1.76, p = 0.079). The difference in Level 3 was not significant anymore.

When considering the time moving towards the right and the left, we observed that, differently from head movements detected in the far space test, for the near space task participants spent on average the same amount of time moving their hand towards both sides (paired t-test; Level1: t = − 0.38, p = 0.702; Level 2: t = − 1.28, p = 0.204; Level 3: t = − 0.60, p = − 0.548).

Finally, from the upper limb tracking data we computed two additional parameters. Overall, the time required to reach a target (time-to-reach) was similar between hemispaces for all levels (paired t-test; Level 1: t = 1.28, p = 0.205; Level 2: t = 0.860, p = 0.393; Level 3: t = 1.258, p = 0.213). Nevertheless, we observed slightly higher reaching times for each of the three columns in the left hemispaces when compared to their counterpart of the right side. These differences reached statistically significance in Level 2 for L1-R1 (Wilcoxon: Z = 3.15, p = 0.002), Level 3 for L2-R2 (t = 2.29, p = 0.025) and Level 3 for L3-R3 (Wilcoxon: Z = 2.45, p = 0.014). Participants’ reaction time was slightly higher for targets appearing in the left hemispace (Wilcoxon sign rank test; Level 1: Z = 3.64, p < 0.001; Level 2: Z = 2.54, p = 0.011; Level 3: Z = 3.01, p = 0.003). This difference was consistently observed in all levels for columns L1-R1 (Level 1: Z = 2.11, p = 0.035; Level 2: Z = 3.89, p < 0.001; Level 3: Z = 1.98, p = 0.048) L2-R2 (Level 1: Z = 3.58, p < 0.001; Level 2: Z = 2.49, p = 0.013; Level 3: Z = 3.26, p = 0.001), and Level 3 for column L3-R3 (Level 1: Z = 1.43, p = 0.152; Level 2: Z = 0.75, p = 0.455; Level 3: Z = 2.72, p = 0.007). Additional file 1: Table S1 shows the detailed scores for each variable of interest.

### Paper-and-pencil tasks

The results of the paper-and-pencil tasks showed similar results as the iVR-based tasks. Regarding the number of omissions in the star cancellation, the average of total omissions in each hemispace was very low, 0.179 (L) and 0.103(R), with no difference between L and R omissions (average L-R omissions difference = 0.077; Wilcoxon sign rank test: Z = 0.905, p = 0.366). 26 participants made their first selection in the left hemispace, as in Level 1 of the far space (statics targets). In the drawing by copy task, the average number of omissions was 0. In the line bisection task, the average bias was of − 0.897 mm.

### Questionnaire

The analysis of the questionnaire scores yielded relevant information about the tasks design and pleasantness/comfort (Table 2). With respect to the questions related to fatigue, relaxation and focus, we found that the iVR-based far space task increased participants’ fatigue (Q1b-Q1) by one point (Wilcoxon sign rank test: Z = − 3.29, p < 0.001); nevertheless, the score post-task remained low (average score < 4), suggesting a moderate disagreement with the presence of tiredness. No significant change in fatigue was observed for the other tasks (all ps > 0.05). Participants reported very high levels of relaxation both before and after the paper-and-pencil and the iVR-based tasks (Q2b-Q2; all scores > 5), with the far space task slightly increasing their level of stress of around half point in the post-task score (Z = 2.41, p = 0.016). Participants remained focused on the tasks (Q3) in all modalities (paper-and-pencil and iVR-based), (average scores > 6). In addition, both iVR-based tasks led to very high level of presence (Q7; average scores > 6), that is the fact of being “inside” the VR and acting on it. In the near space task, a moderate-to-strong feeling of body ownership (Q8; average score = 4.5) and very strong sense of agency (Q9; average score > 6) over the avatar’s arm were elicited.

Importantly, in terms of comfort, the iVR-based tasks did not lead to a substantial physical discomfort (Q10), pain or cybersickness (Q11) due to the HMD (average values ≤ 2.5). Regarding maximal values, two participants reported highest score (7) of dizziness during the near space task, one of them reporting high level (6) as well in the far space task. Regarding the task design, iVR-based tests were defined as slightly more difficult for participants with respect to paper-and-pencil tests, but still easy to perform (Q12; average scores < 3). Moreover, participants agreed that the time-to-reach and target placement were adequately defined (Q13, Q14; average scores > 6). As expected, the auditory dual task slightly increased the difficulty in the far and near space tasks, enough to divide attention but without becoming too disturbing for the main spatial task (Q15, Q16; average scores < 3.5 and < 2.5 for far and near space tasks, respectively). The dual task in the far space task led to a higher level of distraction that the one in the near space task (Z = 3.88, p < 0.001).

In general, all modalities were defined by participants as very enjoyable (Q17) (paper-and-pencil: average score = 6.1; iVR-based tasks: average scores = 5.7 and 4.6 for the far and near space tasks, respectively). Among the three proposed tasks, participants had a clear preference for the iVR-based far space task (the favorite one for 29 out of 39 participants; Q19), which was defined as the more ecological and entertaining.

## Discussion

In this report, we presented two innovative iVR-based assessments for USN, evaluating the far and the near spaces using ecological tasks in multimodal, realistic environments. We have conceived, programmed and tested them in a group of healthy adult participants. The far space task uses head gaze as effector (oculomotor component), while the near space task uses the avatar’s hand (hand-effector) with a radial distribution of targets as it has been shown that neglect patients have a stronger dependency on polar than on cartesian coordinates [36].

As expected, target omissions were very low in all levels of both tasks. For both iVR-based tasks, it seems to be a preference for the left hemispace, as quantified by the exploration time, with that asymmetry being stable across levels. This difference may be partly due to a certain asymmetry of the virtual environment, e.g., similar, but not perfectly mirrored, distribution of ornamental objects, and distribution of targets and distractors distribution in the far space task. Indeed, asymmetric targets and distractors distribution is also present in classical tests like the star cancellation [33]. Other hypotheses include the presence of a pseudoneglect effect, i.e. the natural tendency of shifting spatial attention to the left in healthy individuals (usually present in the bisection line) [37], or the cultural tendency in occidental and right-handed population to explore from left to right that would make participants to spend more time on the left waiting for next target to appear. Independently from the origin of this effect, this left-space prevalence in healthy participants might allow to detect with higher sensitivity spatial attention deficits in left USN patients, which are characterized by the opposite pattern of asymmetry. Importantly, the balance observed in healthy participants’ exploration strategy (time spent moving their heads towards left and right) suggests that the task design is well balanced, with no salient stimuli that could bias attention to a specific side. In association with the omission score, which is a standard parameter to assess asymmetric bias in spatial perception and exploration, we believe that both exploration time and moving time towards left/right may be considered as new sensitive parameters to distinguish neurological patients with and without USN. The combination of both can result in L-R space index ratios as a measure of asymmetry in space exploration.

Exploration times based on the head gaze represent a reliable measure in the far space. To succeed the far space task’s request, a larger exploration on the horizontal plan is required, which cannot be achieved with eye movements only, since targets could appear or occupied near 180° of the space. The assumption that participants may have preferably moved their eyes before their heads to gaze at an object held true for the near space task only. Future developments should thus combine head gaze information with eye tracking to map the exact position of the eyes during the exploration. Eye tracking information can provide further insights on the exploration strategy used by participants, especially in tasks where little or no movements of the head are required, as it is the case for exploration in the peripersonal space [38]. Information about the hand position during the exploration, as collected by the motion tracking system, may provide additional information about motor exploration ratios. Regarding usability, future versions of the solution should include the use of hand controllers, available with most recent HMD, replacing the keyboard to validate user’s actions in the virtual environment. This will represent one more step further toward naturalistic interaction.

Time-to-reach and reaction times provide additional information about motor execution and speed processing, which may be relevant for profiling USN patients. The trial-based design of the task, with participants starting each trial from the body midline position marked by the start pad, might reduce the bias of the arm used. Indeed, we did not observe any increase in time-to-reach when the target was placed on the contralateral side of the hand used.

In our far space task, moving targets approached or drew away from participant’s viewpoint within the same column. Previous iVR-based works included an extrapersonal task to evaluate the ability of street crossing, where participants had to stop the car coming from left or right side before colliding with their avatar [25]. In our task, we decided to quantify the level of “spontaneous” exploration of the full space without having any target or distractor moving in the horizontal plane (i.e., from left to right or vice versa). Moreover, in this study participants were exposed to 360° environments with the possibility of ecologically exploring the scene naturally using the embedded head tracker of the HMD, which was also used to gaze the targets for selection. This is an important advantage with respect to previous iVR-based that limited the field of view (no head tracker active), therefore restricting the possibility of exploration [24, 30], and/or only used a joystick or mouse to select objects in the environment [24, 25, 30].

Questionnaire scores reported good acceptance, low levels of fatigue, and high levels of enjoyment (the far space task was largely preferred). Importantly, both iVR-based tasks were very well tolerated with high levels of comfort (no pain, no dizziness) reported by participants. This is critical as the fear of cybersickness has been reported as a main barrier for the adoption of iVR by clinicians [39]. We also obtained conclusive positive feedback about the quality of the employed technology and the feelings of presence and ownership/agency over the virtual avatar in the iVR-based near space task. All in all, this study thoroughly confirmed the usability of the presented tasks, being one of the critical aspects to be considered when validating new solutions [31].

All the above-described parameters make the present tasks the first multilevel, iVR-based test battery for the assessment of far and near space for both left- and right-sided USN. Indeed, while it has been reported that USN is more frequent and/or more severe after a lesion affecting the right hemisphere, right USN after a left brain damage is also on record [5, 40], and possibly not systematically evaluated with current tools. Our iVR-based battery can be used also to detect a bias in exploration towards the right, and not only the left, hemispace, increasing in the future its clinical utility to the whole stroke population. In both far and near space tasks, although they displayed immersive 3D environments, targets were presented in a 2D plane, either at the floor level in the forest (far) or on the table in the kitchen (near). Additional assessment levels could include 3D arrangements of targets to enable a more precise mapping of deficient attentional spatial areas [36]. A last important clinical aspect is the presence of levels of difficulty, with the possibility to tailor the specific assessment depending on the patient’s characteristics (fatigue, general attentional deficits, severe or very mild neglect). Very important for future clinical applications, the task can be performed at bedside. Finally, our solution includes automated data collection, analysis and storage, which offers additional advantages in terms of reduction of working time for the neuropsychologist and avoiding errors of data scoring.

Our study has several limitations. First, it represents a first-step validation of the formulation, design and implementation of the solution: the clinical validity needs to be corroborated in studies with neurological patients. A first validation report of the far space task in stroke patients can be found in [41]. Second, the sample size was relatively small. Larger samples are needed in order to obtain a dataset for normative data that can be used for diagnostic purposes. Third, as in any technology-mediated assessment, although the design of the task is hardware-agnostic, an impact of different hardware supports cannot be excluded: therefore, the robustness of the assessment in this regard should be verified before its market entry.

To conclude, the presented assessment protocols seem a useful and well-tolerated tool to assess visuo-motor and visuo-perceptual exploration. They could also be used as preliminary examinations to detect selective defective cognitive components, and even to implement and personalize therapy protocols within a clinical setting.

## Supplementary Information

**Additional file 1: Table S1.** Scores (mean ± std) of the parameters recorded for both the iVR-based far and near space tasks. Time is provided in seconds. Percentages and number of omissions are provided in brackets.

## Data Availability

Data are available from the corresponding authors upon reasonable request and with permission of MindMaze.
