# Supplementary material for: An immersive virtual reality system for ecological assessment of peripersonal and extrapersonal unilateral spatial neglect
Source: J Neuroeng Rehabil. 2023 Mar 18;20:33. doi: 10.1186/s12984-023-01156-1 (PMC10024837; doi:10.1186/s12984-023-01156-1)
Supplement: Supplementary file 1 — Additional file 1: Table S1. Scores (mean ± std) of the parameters recorded for both the iVR-based far and near space tasks. Time is provided in seconds. Percentages and number of omissions are provided in brackets. [file 12984_2023_1156_MOESM1_ESM.docx]

**An Immersive Virtual Reality System for Ecological Assessment of Peripersonal and Extrapersonal Unilateral Spatial Neglect**

**Table S1.** Scores (mean±std) of the parameters recorded for both the iVR-based far and near space tasks. Time is provided in seconds. Percentages and number of omissions are provided in brackets.

|  | Far space task | | | | Near space task | | |
| --- | --- | --- | --- | --- | --- | --- | --- |
|  | Level 1 | Level 2 | Level 3 | Level 4 | Level 1 | Level 2 | Level 3 |
| Omitted targets L  [min, max] | 0.1±0.3  [0, 1] | 0.5±0.7  [0, 2] | 0.3±0.5  [0, 2] | 0.3±0.6  [0, 2] | 0.1±0.5  [0, 3] | 0.1±0.3  [0, 1] | 0.0±0.2  [0, 1] |
| Omitted targets R  [min, max] | 0.1±0.3  [0, 1] | 0.7±0.9  [0, 4] | 0.9±0.8  [0, 2] | 0.4±0.6  [0, 2] | 0.1±0.3  [0, 1] | 0.4±0.6  [0, 2] | 0.2±0.5  [0, 2] |
| Omitted targets L-R  [min, max] | 0.0±0.3  [-1, 1] | -0.2±1.2  [-4, 2] | -0.6±0.8  [-2, 1] | -0.2±0.7  [-2, 1] | 0.0±0.4  [-1, 2] | -0.2±0.5  [-2, 1] | -0.1±0.4  [-2, 0] |
| Time moving towards L (%) | 29.8±8.8  (50.7±4.0) | 241.5±20.0  (48.8±2.2) | 290.2±19.4  (50.9±2.2) | 276.2±17.2  (50.9±2.3) | 226.1±33.1  (49.6±3.1) | 215.2±29.8  (49.0±3.2) | 220.2±32.5  (49.5±3.6) |
| Time moving towards R (%) | 29.1±8.6  (49.3±4.0) | 253.1±16.2  (51.2±2.2) | 279.7±18.6  (49.1±2.2) | 266.2±14.0  (49.1±2.3) | 228.8±26.5  (50.4±3.1) | 223.6±26.1  (51.0±3.2) | 225.0±35.0  (50.5±3.6) |
| Total exploration time | 59.0±16.8 | 494.6±29.1 | 569.9±28.5 | 542.4±19.3 | 455.0±52.6 | 436.9±48.9 | 443.3±60.2 |
| Exploration time L  (%) | 29.7±9.4 (50.4±7.4) | 277.2±44.2 (56.0±7.5) | 313.3±35.4 (55.0±5.8) | 293.6±33.3 (54.2±6.0) | 289.9±81.4 (63.5±16.0) | 292.4±88.5 (66.8±19.0) | 252.9±89.0 (57.3±19.9) |
| Exploration time R  (%) | 29.3±9.8 (49.6±7.4) | 217.3±35.6 (44.0±7.4) | 256.5±36.5 (45.0±5.8) | 248.7±34.2 (45.8±6.0) | 164.9±69.6 (36.5±16.0) | 144.3±78.7 (33.2±18.9) | 190. ±98.4 (42.6±19.8) |
| Exploration time L-R  (%) | 0.4±9.5 (0.8±14.9) | 59.9±74.8 (11.9±14.9) | 56.8±66.1 (10.0±11.7) | 44.9±64.6 (8.3±11.9) | 125.0±142.1  (27.0±32.0) | 148.1±160.2 (33.6±37.9) | 62.6±177.6 (14.7±39.7) |
| Ratio of exploration time L | 3.3±1.0 | 15.8±2.5 | 17.7±2.0 | 16.6±2.0 | 8.1±2.3 | 8.1±2.5 | 7.0±2.5 |
| Ratio of exploration time R | 3.3±1.1 | 12.6±2.0 | 15.0±2.3 | 14.2±2.1 | 4.6±1.9 | 4.0±2.2 | 5.3±2.7 |
| Ratio of exploration time L-R | 0.0±1.1 | 3.2±3.9 | 2.7±3.7 | 2.4±3.8 | 3.5±4.0 | 4.1±4.5 | 1.7±4.9 |
| Marking distance L | 12.5±2.3 |  |  |  |  |  |  |
| Marking distance R | 12.2±2.2 |  |  |  |  |  |  |
| STD Marking distance L | 11.7±2.2 |  |  |  |  |  |  |
| STD Marking distance R | 11.4±2.1 |  |  |  |  |  |  |
| Marking time L | 2.6±1.2 |  |  |  |  |  |  |
| Marking time R | 2.5±1.2 |  |  |  |  |  |  |
| STD Marking time L | 1.6±1.5 |  |  |  |  |  |  |
| STD Marking time R | 1.6±1.4 |  |  |  |  |  |  |
| Time-to-reach L |  |  |  |  | 1.8±0.3 | 1.8±0.3 | 1.8±0.2 |
| Time-to-reach R |  |  |  |  | 1.8±0.2 | 1.7±0.3 | 1.7±0.2 |
| Time-to-reach L1 |  |  |  |  | 1.7±0.3 | 1.7±0.2 | 1.6±0.2 |
| Time-to-reach R1 |  |  |  |  | 1.6±0.2 | 1.6±0.2 | 1.6±0.2 |
| Time-to-reach L2 |  |  |  |  | 1.9±0.2 | 1.8±0.3 | 1.8±0.2 |
| Time-to-reach R2 |  |  |  |  | 1.8±0.2 | 1.7±0.3 | 1.7±0.2 |
| Time-to-reach L3 |  |  |  |  | 2.0±0.4 | 1.9±0.3 | 1.9±0.2 |
| Time-to-reach R3 |  |  |  |  | 1.9±0.3 | 1.9±0.4 | 1.8±0.3 |
| Reaction time L |  |  |  |  | 0.8±.01 | 0.8±0.1 | 0.8±0.1 |
| Reaction time R |  |  |  |  | 0.8±0.1 | 0.8±0.1 | 0.7±0.1 |
| Reaction time L1 |  |  |  |  | 0.8±0.1 | 0.8±0.1 | 0.7±0.1 |
| Reaction time R1 |  |  |  |  | 0.7±0.1 | 0.7±0.1 | 0.7±0.1 |
| Reaction time L2 |  |  |  |  | 0.8±0.1 | 0.8±0.1 | 0.7±0.1 |
| Reaction time R2 |  |  |  |  | 0.7±0.1 | 0.7±0.1 | 0.7±0.1 |
| Reaction time L3 |  |  |  |  | 0.8±0.2 | 0.8±0.1 | 0.8±0.1 |
| Reaction time R3 |  |  |  |  | 0.8±0.2 | 0.8±0.2 | 0.8±0.2 |
